# Constitution Building

**Published:** 1888-05-05

**Authors:** 


### The Doctor's Pulpit.

### CONSTITUTION BUILDING.

Nature gives high rewards to capable constitution builders. She gives such impetus and help in all directions that, provided the start has been good, and the work of the builder fairly well directed, accidents apart, the result is sure to be successful. Skilful farmers and stockbreeders know this perfectly well. The difference between the fields and the cattle of the competent agriculturist and those of the stupid boor is amazing. It is curious and disappointing that more pains and skill should be ordinarily expended cn cattle, turnips, and wheat than on the management and rearing of children. Cattle and turnips have a marketable value, say some; but have not children a value which no market can fix or even guess at? Many mothers display a careless indifference, a want of painstaking thoroughness in the management of their children, which is almost incredible. It is not that they do not love them, but it is that they do not appear to have the intellectual capacity to understand what is required to be done for them, and to do it. Topsy "'spected she growed." Many children, even in the most highly-civilised communities, and among those who have the greatest possibilities of cultured intelligence, merely "grow," just as much as Topsy did, though in a different way. A witty Lancashire operative, who had known rough usage in his boyhood, was once asked where he was "brought up." He answered, "I never was brought up. I was *kicked up*."

Fathers, it is to be feared, do not always take their full share in the early management of their children. They think the period of infancy belongs of right to the mother. Perhaps in some respects it does. But the responsibility of bringing up boys and girls from the very date of birth appertains to both parents, and the one whose duty it is to bear the fullest weight of it is not necessarily the mother at any particular stage or the father at any particular stage. The rule should be that the parent who has the better capacity should accept the more responsibility. At the same time, the period cf infancy does most naturally belong to the mother. But early infancy was discussed last week, and now a little space may be devoted to boyhood and girlhood. At this stage at any rate the father will admit the justice of the claims which may be made upon him; and if his business be such as to tax all his energies, he must at least give the mother his intelligent sympathy, and such occasional help as health and other circumstances will permit.

Childhood presents different problems from those of infancy. Not only do the food and clothing differ, but the question of education is added, and also that of moral training. The order of importance, however, in early childhood must still be the bodily health first. For it cannot be too clearly understood that the physical is the basis of both the mental and the moral. Other things being equal, the healthiest child will be the cleverest and also the most docile and virtuous. Disobedience, peevishness, wilfulness, laziness, are often mere outward indications of a disordered stomach or liver. A powder is frequently the proper corrective for a fit of ill-temper; an alterative or a tonic the true cure for a peevish disposition. Of course, this is not always the case. Children may be naughty, and require both reproof and the cane; but the present writer has very great faith in a sound digestion and well-regulated bowels.

The feeding of boys and girls is as important as the feeding of infants. Certain rules should be observed, and among those rules this should stand first: Each child must be fed according to his own necessities. Many mothers have a Mede and Persian code of regulations transmitted from remote ancestors, which is the bane of child-life. Some Scotch people set an absurd store by their national porridge. Porridge is an excellent thing, and for fifteen children in every twenty may be one of the best foods possible. But some children detest porridge. They cannot be made to take it without the greatest trouble. In such cases it is probable they would be much better without it.

In England, again, many people are afraid of meat for their children. "Puddings, puddings, puddings, and milk, milk, milk!" is still the burden of their cry. Now both puddings and milk are admirable for many children, but surely it may be understood that they are not good or suitable for all. Some children require a little good meat once or twice daily, or fresh eggs, or beef-tea, or veal broth, or soups of various kinds. The proper course is to watch them intelligently; to consult a doctor who takes an interest in such questions; to observe the effects of different kinds of diet for a definite period; and to use that kind of dietary which experience proves to give the best results. But all this implies thought, care, labour, and intelligence, you say. Certainly; and what is our intelligence given for if not to use on worthy objects; and what possible object can be more worthy of our best intelligence and labour than the building up of the fabric of body, mind, and character in our own children?

The clothing of boys and girls in our climate requires consideration. Boys are easily managed. Let them have flannel from head to foot; thin in summer and thicker in winter; and let their other garments be light and loose, and they will mostly be well protected against variable weather. The very same rules apply to girls; but fashion which is an abomination to every intelligent doctor, comes in and entirely upsets all our calculations. Why will mothers clothe their children in short frocks—loose and low round the neck—short stockings which leave a portion of the legs bare, tight corsets, and other such villainous contrivances? Even women might understand that we have in this country a climate, which though bracing and healthy for the strong, is most fickle and dangerous for the weak. The climate is a hard fact which nobody can alter. Fashion is a wretched and changeable fetish, more contemptible than the bull worshipped by the ancient Egyptians, or the piece of carved wood adored by the South Sea Islanders. The writer has a profound regard for the nobler qualities of women, but an inexpressible contempt for some of their miserable weaknesses. This language is strong; but when a man's only way of compelling women to think is the method of the pen, he must use such words as are likely to effect his purpose. If a woman is determined to risk her own health and life at the dictates of fashion, she might at least have enough of pity and kindness of heart to restrain her from destroying the health and life of her children.

Education is a disturbing element in the building of the human constitution. When the farmer rears his cattle and horses he has no care for their education. He secures a healthy stock to begin with, and then places the young in the healthiest possible conditions, and leaves the rest to the generous hand of nature. But with human beings the schoolmaster has long been an opposing force; and of late years the examiner has appeared, to make matters ten times worse. The system of examination pursued in elementary schools of all classes is an almost unmitigated evil. It is an evil from the educational standpoint; but it is infinitely more so from the medical. What is education? Is it cram, or is it the training of the mind? That is the real question at issue. Even from the purely utilitarian point of view the man whose mind is trained, though his knowledge facts be smaller, will almost always beat the crammed man in the pursuits of actual life. Our present methods school teaching and examining do infinite harm to the bodily health of boys and girls, and in many cases almost as much harm to their minds. Education, conducted as it should be, is an aid to physical development; but education which exhausts, worries, diminishes the sleep, keeps the pupil a long time in a vitiated atmosphere, and unfits him for abundance of self-forgetting play, is an injury in every way, and often a fatal mistake. Up to a certain point, bodily exercise promotes the growth and strength of the body; beyond that point it is injurious. It is precisely so with the brain. Within ascertainable limits every boy and girl is physically as well as intellectually and morally improved by study and education. Outside those limits there is harm, and only harm, in every direction. We want in this country an Education Department, with a man of true statesmanlike capacity at its head; and under him we want a system of teaching which shall teach and not cram, and methods of examining which shall test the general mental capacity, not the single faculty of memory. This implies both more work and more intelligence in high quarters. Does experience warrant us in expecting them? In any case, individual parents must resolve that on no account shall any system of education rob their children of that bodily and mental health without which the highest scholastic attainments and the finest opportunities are alike powerless to produce either happiness or success.

